# Supplementary figures and images for: Does Delaying Time in Cancer Treatment Affect Mortality? A Retrospective Cohort Study of Korean Lung and Gastric Cancer Patients
Source: Int J Environ Res Public Health. 2021 Mar 26;18(7):3462. doi: 10.3390/ijerph18073462 (PMC8036321; doi:10.3390/ijerph18073462)

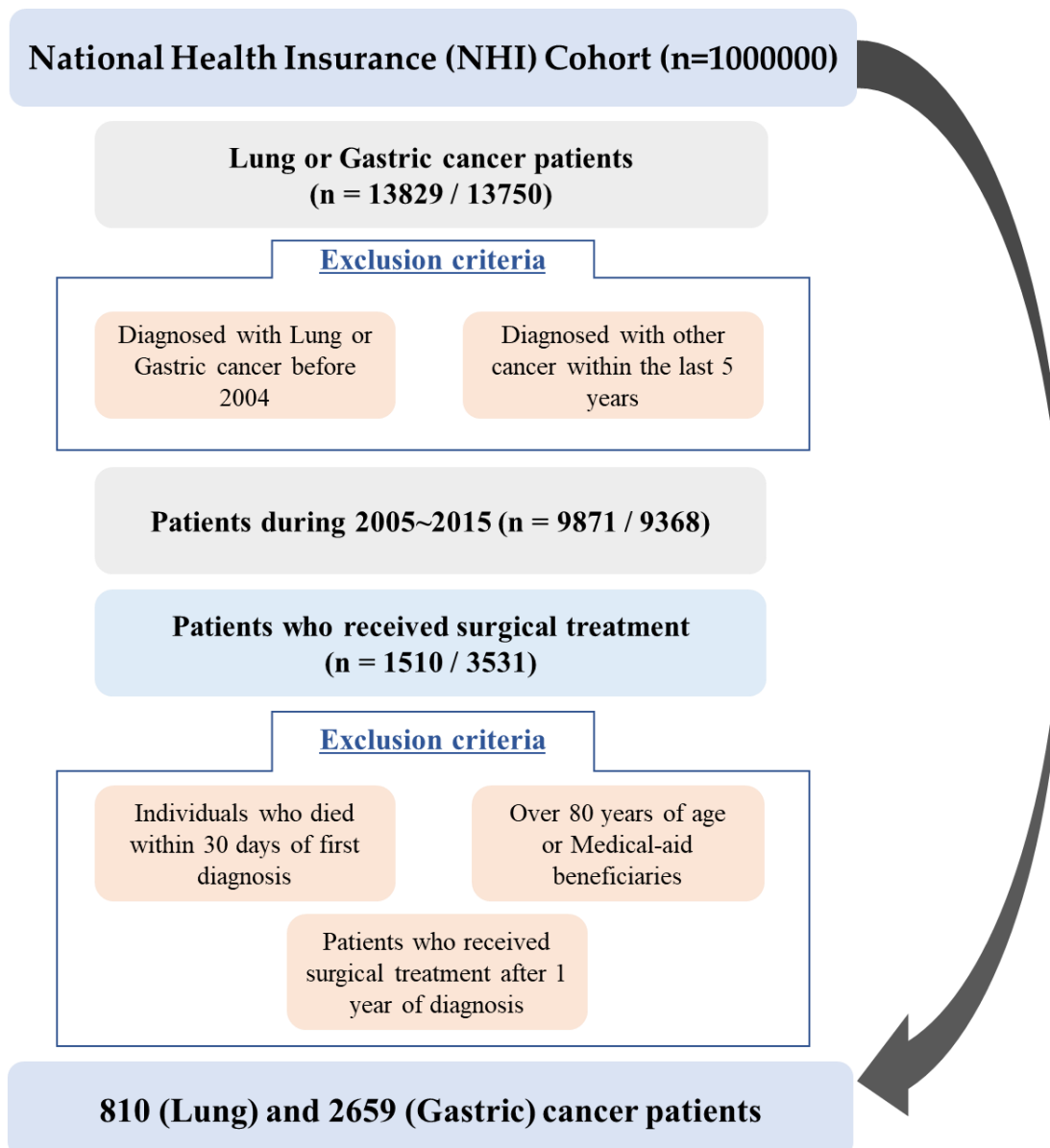

Figure S1. Flow diagram of study participants

Supplement: Supplementary file 1 [file ijerph-18-03462-s001.pdf]
